# Correction: X-ray absorption spectroscopy of exemplary platinum porphyrin and corrole derivatives: metal- *versus* ligand-centered oxidation

**DOI:** 10.1039/d1ra90156g

**Published:** 2021-10-11

**Authors:** Benjamin D. Matson, Kolle E. Thomas, Abraham B. Alemayehu, Abhik Ghosh, Ritimukta Sarangi

**Affiliations:** Stanford Synchrotron Radiation Lightsource, SLAC National Accelerator Laboratory, Stanford University Menlo Park California 94025 USA ritis@slac.stanford.edu; Department of Chemistry, UiT – The Arctic University of Norway N-9037 Tromsø Norway abhik.ghosh@uit.no

## Abstract

Correction for ‘X-ray absorption spectroscopy of exemplary platinum porphyrin and corrole derivatives: metal- *versus* ligand-centered oxidation’ by Benjamin D. Matson *et al.*, *RSC Adv.*, 2021, **11**, 32269–32274. DOI: 10.1039/D1RA06151H

The authors regret that the one of the author affiliations was incorrectly shown in the original manuscript. The corrected list of affiliations is as shown above.

The Royal Society of Chemistry apologises for these errors and any consequent inconvenience to authors and readers.

## Supplementary Material

